# Ten-year longitudinal investigation of astigmatism: The Yamagata Study (Funagata)

**DOI:** 10.1371/journal.pone.0261324

**Published:** 2022-01-10

**Authors:** Hiroyuki Namba, Akira Sugano, Takanori Murakami, Hiroshi Utsunomiya, Hidenori Sato, Koichi Nishitsuka, Kenichi Ishizawa, Takamasa Kayama, Hidetoshi Yamashita

**Affiliations:** 1 Faculty of Medicine, Department of Ophthalmology and Visual Sciences, Yamagata University, Yamagata City, Yamagata, Japan; 2 Ideganka Hospital, Yamagata City, Yamagata, Japan; 3 Department of Ophthalmology, Yamagata Prefectural Central Hospital, Yamagata City, Yamagata, Japan; 4 Faculty of Medicine, Genome Informatics Unit, Institute for Promotion of Medical Science Research, Yamagata University, Yamagata City, Yamagata, Japan; 5 Faculty of Medicine, Department of Neurology, Hematology, Metabolism, Endocrinology and Diabetology, Yamagata University, Yamagata City, Yamagata, Japan; 6 Faculty of Medicine, Department of Advanced Medicine, Yamagata University, Yamagata City, Yamagata, Japan; Saarland University, GERMANY

## Abstract

Despite numerous investigations into ocular or corneal astigmatism, the dynamic nature of astigmatism remains poorly understood. To reveal potential associations between age and astigmatism, 264 Japanese participants who underwent systemic and ophthalmological examinations in Funagata Town (Yamagata Prefecture, Japan) were evaluated over a 10-year period. Astigmatism was evaluated with regard to the cylinder power, cylinder axis, and vector analyses. Whereas the refractive cylinders showed age-related increases in patients in their 40s to 60s, the corneal cylinders did not change over 10 years. Nevertheless, cylindrical axis of the cornea demonstrated a continuous shift toward against-the-rule (ATR) astigmatism. Vector analyses revealed that the astigmatic shift toward ATR progressed continually after patients reached their 40s, although the shift did not accelerate with age. These novel insights may pave the way for the development of potential strategies for vision correction, including refractive surgeries, and vision-quality maintenance in the elderly.

## Introduction

The human eye functions as an optical system that focuses visual images onto the retina. Astigmatism lowers the vision quality by distorting images and causing visual disturbances and blurring in uncorrected or corrected eyes [1–3]. Previous findings have indicated that anterior corneal astigmatism is compensated for by internal optics, and in part, by the posterior corneal surface [4,5]. Previous data have also shown that the prevalence of astigmatism increases [6–9] and that the axes of astigmatism shift from with-the-rule (WTR) toward against-the-rule (ATR) with aging [10–17]. However, the underlying mechanisms and processes remain poorly understood. It has been reported that analyzing data separately regarding the magnitude and axis of astigmatism is insufficient to assess the dynamic changes in astigmatism. The dynamic nature of astigmatism has been investigated using graphical vector analysis [18], the polar value method [19], and power vector analysis [20]. In present study, we addressed this problem by vector analysis using the Alpins method. [21–23] Optical deterioration with age is expected to cause severe problems in aging societies. The purpose of this study was to investigate astigmatic changes in Japanese adults over a period of 10 years. From the perspective of optical corrections, including surgical procedures, a better understanding of the causes of astigmatism may reduce the likelihood of unfavorable results and should help in developing improved strategies for maintaining vision quality in the elderly.

## Methods

### Subjects

This study was performed as part of the Yamagata Study (Funagata), a population-based epidemiologic study examining systemic and ophthalmologic disorders in adult Japanese individuals aged 35 years and older. Details regarding the study participants and the research methods used were described previously [17,24–30]. Systemic and ophthalmic data were obtained from residents living in Funagata Town once every 5 years. Results obtained from 2005 to 2007 (phase 1), from 2010 to 2012 (phase 2), and from 2015 to 2017 (phase 3) were examined. Before the examinations started, written informed consents were obtained from all study participants. This study adhered to the tenets of the Declaration of Helsinki. The Yamagata Study (Funagata) was approved by the Ethical Review Committee of Yamagata University Faculty of Medicine, Yamagata, Japan. Patients were excluded from this study if they had a history of any ocular surgery, corneal scarring, other corneal pathology (e.g. pterygium), or wearing contact lenses upon slit-lamp examination. Patients who had conditions or a history of surgeries that could potentially influence astigmatism were excluded from this study, based on information gathered when obtaining patient medical histories. Subjects with missing or insufficient data were also excluded. The subjects were divided into five age groups, i.e. ≤ 39 years, 40–49 years, 50–59 years, 60–69 years, and ≥ 70 years at the beginning of phase 1.

### Examination

The refractive spherical and cylindrical power, corneal cylindrical power, and intraocular pressure were measured with an auto-ref/kerato/tonometer (TONOREF II, Nidek Co., Ltd., Aichi, Japan). Refractive and corneal astigmatisms were defined as being WTR when the steepest meridian was within 90 ± 30°, or as being ATR when the steepest meridian was from 0 to 30° and 150 to 180°. Otherwise, the astigmatisms were defined as being oblique. The axial length was measured by performing partial-coherence laser interferometry using an OA-1000 optical biometer (TOMEY Corp., Aichi, Japan). Only the data obtained from the right eye of each individual were included to avoid the influence of interdependence between eyes. Physical characteristics, such as height and weight, were measured while subjects were wearing light clothing, without shoes. To avoid the use of interdependent data between two eyes from the same subject, data from only the right eyes were used.

### Vector analysis

As described in previous reports [22], the refractive and keratometric astigmatism, at phase 1 and 3, were plotted on single-angle polar diagrams. Vector differences between phase 1 and 3 were also plotted. Additionally, double-angle vector investigations were performed to estimate orthogonal astigmatism. The polar values of the refractive and keratometric astigmatism were calculated by the following equation on the double-angle diagram:

Polarvalue=Kcos(180‐2θ)

where K represents the refractive or keratometric cylinder magnitude (D, diopters), and **θ** represents the steepest meridian (degree). Positive polar values indicate WTR astigmatism, whereas negative polar values indicate ATR astigmatism.

### Statistical analyses

The data were analyzed using statistical SPSS analysis software, version 21.0 (https://www.ibm.com/analytics/spss-statistics-software, IBM Corp, Armonk, NY, USA). P-values less than 0.05 were considered to reflect significant differences. In cross-sectional investigation, the difference in astigmatism magnitudes and polar values of each age group were examined by Kruskal–Wallis tests. When performing longitudinal investigations of astigmatism, time-dependent changes were estimated by Friedman tests. Changes in polar values between phase 1 and 2, and between phase 2 and 3 were compared by performing Mann-Whitney tests. Linear regression analyses were performed in investigating the association between baseline age and change magnitude of astigmatism over 10 years.

## Results

### Demographic characteristics

The ophthalmological examinations included 1114 patients in phase 1. In those patients, 783 patients also attended in phase 2, and the 329 patients participated in all of the phase 1 to 3. Forty-five patients were excluded for the reason of pseudophakia, pterygia, or other abnormalities. From the mean value and standard deviation of spherical equivalent, -0.97 ± 2.31 diopters (D), we excluded additional 20 patients whose spherical equivalent were less than -5.59 D, and more than 3.64 D. As a result, data from 264 subjects (118 men and 146 women) were available for this study. The characteristics are summarized in Table 1. The mean age was 56.9 ± 8.9 years.

**Table 1:** Patient demographics.

	N (%)	Mean	Standard deviation
Age (years)		56.9	8.9
35–39	9 (3.4)		
40–49	44 (16.7)		
50–59	118 (44.7)		
60–69	71 (26.9)		
70–79	22 (8.3)		
Sex			
Male	118 (44.7)		
Female	146 (55.3)		
Height (cm)		157.2	9.5
Weight (kg)		59.7	11.3
BMI (kg/m^2^)		24.1	3.3
IOP (mmHg)		12.7	2.9
Axial length (mm)		23.5	1.1

Abbreviations: BMI, body mass index; IOP, intraocular pressure.

### Astigmatism magnitude and meridian

**Fig 1:**
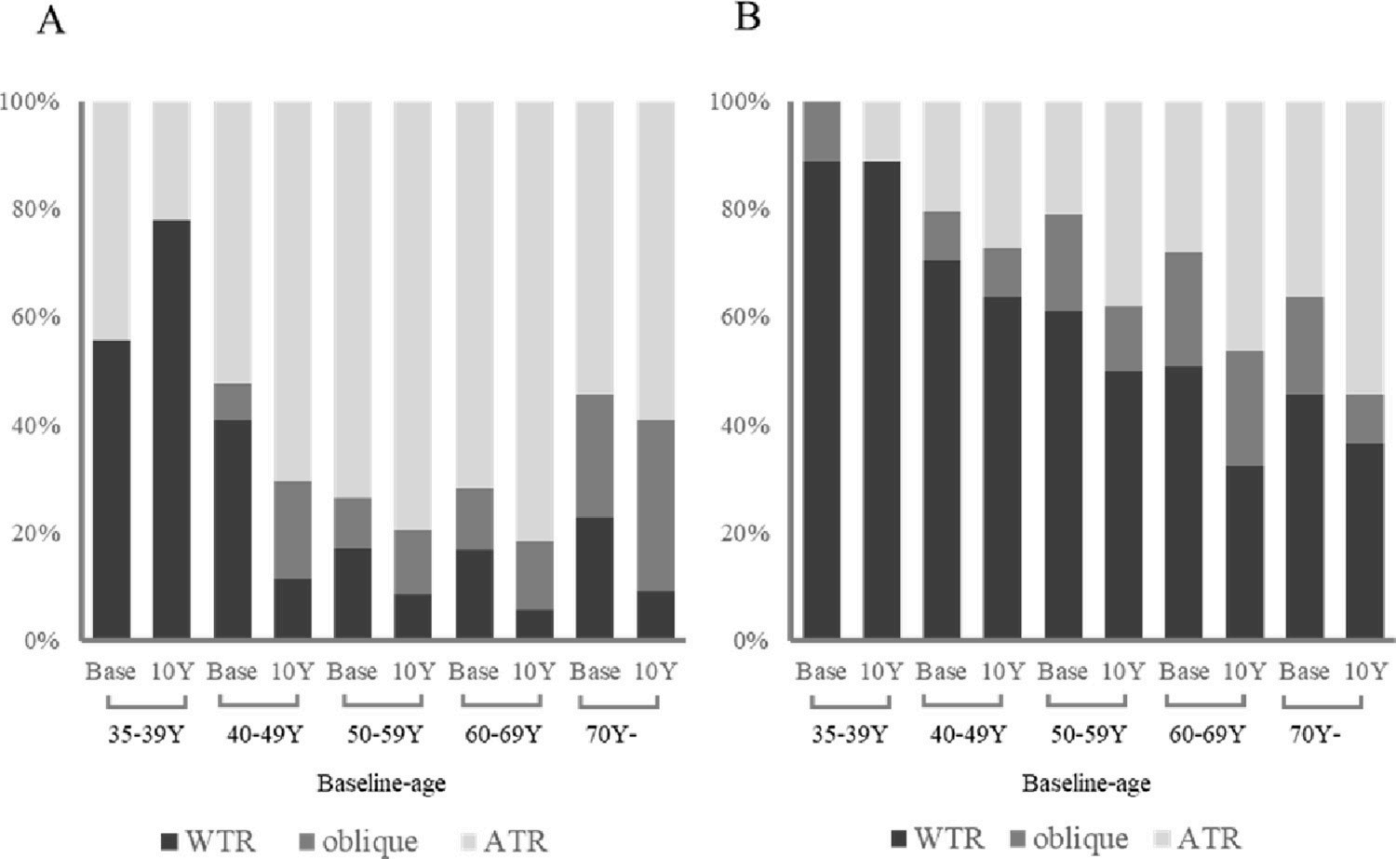
Time-dependent changes in the distribution of the astigmatism during phase 1 (baseline; Base) and phase 3 (10 years later; 10 Y) among the different age groups. In accordance with the meridians, subjects were divided into three astigmatism groups, namely the WTR, oblique, and ATR groups. (**A**) Changes in refractive astigmatism. (**B**) Changes in keratometric astigmatism.

Table 2 demonstrates age-related variations and time-dependent changes that occurred in the refractive cylinder (RC) and keratometric cylinder (KC) values. Cross-sectional investigation showed that, although RC values increased in phase 3, the other values did not show certain trends. Similarly, longitudinal investigation from phase 1 to 3 showed that KC values did not change over the 10-year period, although RC values increased in three age groups (40–49, 50–59, and 60–69 years). Fig 1 shows the time-dependent changes in the cylindrical axis distribution during phase 1 (baseline) and phase 3 (10 years later). Fig 1A shows the changes in the refractive axis, with the axis changing rapidly toward ATR in subjects in their 40s and 50s. Investigating corneal astigmatisms (Fig 1B) revealed a continuous shift toward ATR, except in the youngest group.

**Table 2:** Age-related variations and time-dependent changes in the refractive and keratometric cylinder magnitudes.

	Age group	Phase 1		Phase 2		Phase 3		P-value, Friedman test
		Mean	SD	Mean	SD	Mean	SD	
RC (D)	Total	0.75	0.59	0.98	0.70	1.06	0.69	< 0.001
	35–39	0.83	0.50	0.81	0.53	0.67	0.50	0.497
	40–49	0.63	0.59	1.05	0.85	0.86	0.66	0.001
	50–59	0.69	0.51	0.90	0.59	0.99	0.60	< 0.001
	60–69	0.87	0.68	1.04	0.69	1.31	0.80	< 0.001
	≥70	0.95	0.68	1.13	0.92	1.11	0.68	0.607
	P-value, Kruskal–Wallis test	0.095		0.724		0.002		
KC (D)	Total	0.76	0.71	0.75	0.56	0.72	0.56	0.367
	35–39	0.92	0.45	0.92	0.53	1.08	0.91	0.670
	40–49	0.75	0.58	0.81	0.65	0.69	0.53	0.236
	50–59	0.60	0.42	0.67	0.49	0.65	0.47	0.293
	60–69	0.90	1.01	0.78	0.60	0.76	0.54	0.945
	≥70	1.10	0.92	0.85	0.64	0.84	0.84	0.348
	P-value, Kruskal–Wallis test	0.032		0.469		0.391		

Abbreviations: SD, standard deviation; RC, refractive cylinder; KC, keratometric cylinder; D, diopters.

### Investigating astigmatism

**Fig 2:**
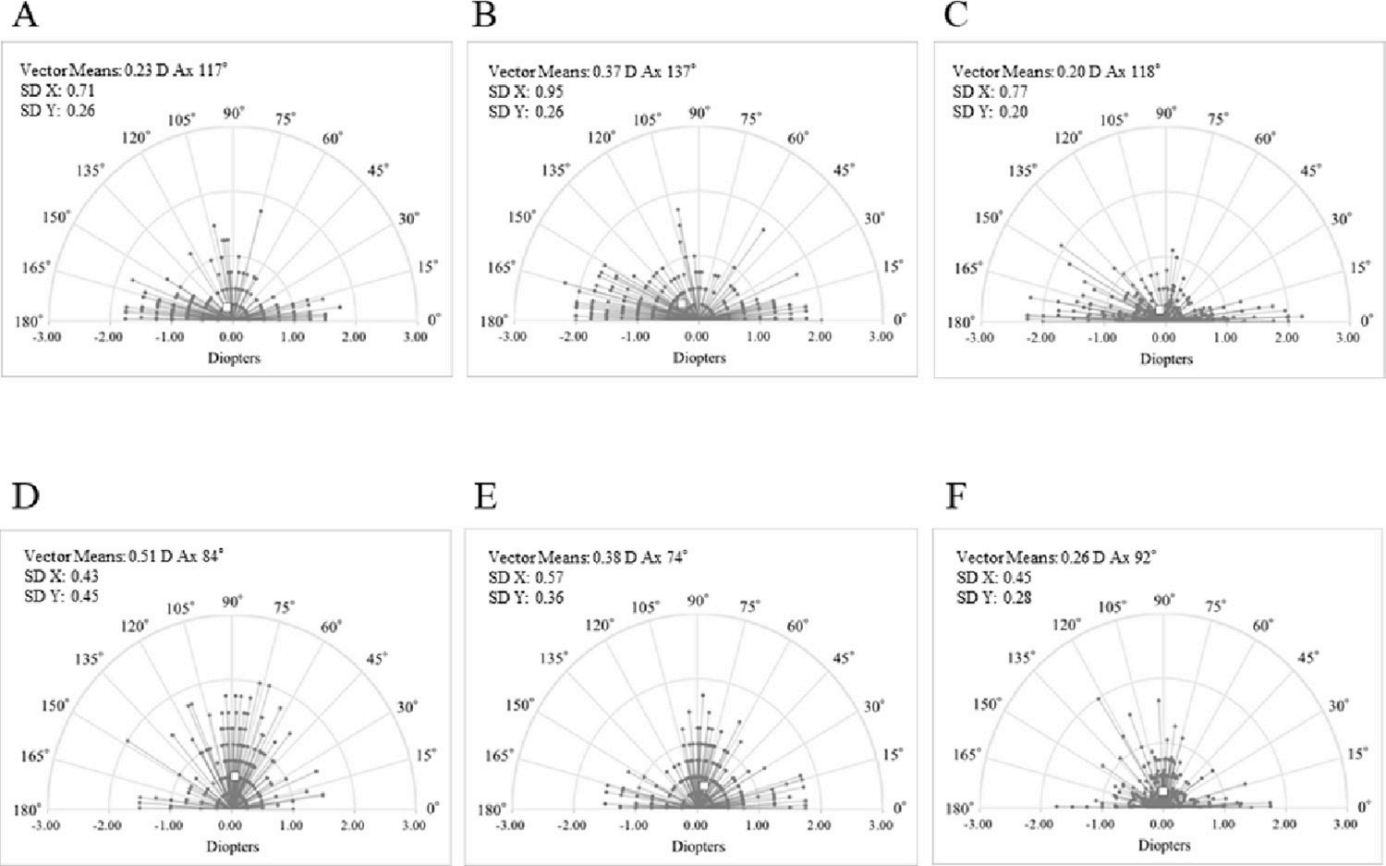
Single-angle polar plots of refractive and keratometric astigmatism. (A) Refractive astigmatism in phase 1. (B) Refractive astigmatism in phase 3. (C) Refractive astigmatism difference between phase 1 and 3. (D) Keratometric astigmatism in phase 1. (E) Keratometric astigmatism in phase 3. (F) Keratometric astigmatism difference between phase 1 and 3. White box in each diagram indicates vector mean.

As described previously [21–23] we described refractive and keratometric astigmatism on single-angle polar plots in accordance with Alpins method (Fig 2). The mean refractive cylinder was 0.23 D at an axis of 117° in phase 1, and 0.37 D at an axis of 137° in phase 3. The mean keratometric astigmatism was 0.51 D at an axis of 84° in phase 1, and 0.8 D at an axis of 74° in phase 3. The vector differences were 0.20 D at an axis of 118° and 0.26 D at an axis of 92°, respectively.

To investigate polar change over 10 years, polar analyses in double-angle plots were examined. Table 3 summarizes the mean polar values in each phase calculated as above. The results for each age group are also presented. The keratometric polar values significantly decreased in a time-dependent manner in subjects aged 40–49 (p < 0.001), 50–59 (p < 0.001), and 60–69 years (p < 0.001), as determined by Friedman tests. These data indicate that a time-dependent shift toward ATR occurred. However, the refractive and keratometric polar values did not differ significantly in the group of subjects aged ≤ 39 and ≥ 70 years.

**Table 3:** Age-related variations and time-dependent changes in the refractive and keratometric polar values.

	Age group	Phase 1		Phase 2		Phase 3		P-value, Friedman test
		Mean	SD	Mean	SD	Mean	SD	
Refractive polar values (D)	Total	-0.39	0.61	-0.51	0.71	-0.63	0.71	< 0.001
	35–39	0.23	0.75	0.03	0.89	0.10	0.59	0.412
	40–49	-0.32	0.55	-0.41	0.64	-0.53	0.60	0.002
	50–59	-0.43	0.50	-0.58	0.62	-0.73	0.63	< 0.001
	60–69	-0.54	0.55	-0.63	0.58	-0.82	0.68	0.001
	≥70	-0.37	0.74	-0.37	0.74	-0.52	0.64	0.943
P-value, Kruskal–Wallis test		0.121		0.023		0.001		
Keratometric polar values (D)	Total	0.25	0.63	0.06	0.67	-0.04	0.68	< 0.001
	35–39	0.87	0.47	0.50	0.75	0.59	0.44	0.156
	40–49	0.45	0.63	0.28	0.67	0.12	0.66	< 0.001
	50–59	0.27	0.52	0.14	0.60	0.02	0.60	< 0.001
	60–69	0.11	0.54	-0.03	0.60	-0.14	0.61	< 0.001
	≥70	0.02	0.53	-0.07	0.70	-0.23	0.51	0.099
P-value, Kruskal–Wallis test		0.004		0.060		0.007		

Abbreviation: SD, standard deviation; D, diopters.

Change in the polar values between phase 1 and 2, and between phase 2 and 3 are summarized on Table 4. Any polar values did not change between the two periods except for the refractive value of subjects aged ≤ 39, indicating that astigmatic shift toward ATR did not accelerate with age.

**Table 4:** Comparisons of changes in the polar values between two periods.

	Age group	Phase 1 to phase 2		Phase 2 to phase 3		P-value, Mann-Whitney U test
		Mean	SD	Mean	SD	
Refractive polar changes (D)	Total	-0.12	0.59	-0.15	0.56	0.303
	35–39	-0.20	0.83	0.08	0.76	0.040
	40–49	-0.09	0.56	-0.11	0.51	0.856
	50–59	-0.15	0.56	-0.15	0.54	0.463
	60–69	-0.09	0.51	-0.19	0.53	0.446
	70–79	0.00	1.02	-0.14	0.81	0.663
Keratometric polar changes (D)	Total	-0.15	0.52	-0.12	0.55	0.587
	35–39	-0.37	0.71	0.08	0.70	0.694
	40–49	-0.18	0.50	-0.16	0.59	0.829
	50–59	-0.13	0.52	-0.12	0.54	0.966
	60–69	-0.14	0.53	-0.11	0.53	0.540
	70–79	-0.10	0.44	-0.16	0.47	0.597

Abbreviations: SD, standard deviation; D, diopters.

## Discussion

In this study, we investigated associations of astigmatism with age. Three main findings resulted from this work, namely, (i) KC values did not increase with age, although RC values did; (ii) the astigmatic shifts toward ATR were seen from 40s to 60s; and (iii) the astigmatic shift toward ATR did not accelerate with age.

Previous studies focused on refractive astigmatism showed that the prevalence of astigmatism increased with age in adult subjects [6–9]. Similarly, in our cross-sectional and longitudinal investigations, RC values showed age-related increases in subjects in their 40s, 50s, and 60s, indicating that the astigmatic magnitude increased in the whole eye. Nevertheless, KC values did not change with aging. The astigmatic magnitude of the cornea appeared to be stable. Similarly, Sanfilippo *et al*. [12] showed that the prevalence of refractive astigmatism increases with age after the age 50 years, although corneal astigmatism was stable until the age of 80. Herein, the age-related changes in the astigmatic meridians were also estimated (Fig 1). Although astigmatic meridians of the cornea and whole eye appeared to change with age, they shifted differently. The keratometric meridian shifted continuously toward ATR, whereas the refractive meridian changed rapidly toward ATR in subjects in their 40s and 50s. Therefore, corneal astigmatism appears to shift toward ATR without an increase in the cylinder power, whereas the cylinder power of the crystalline lens appears to increase with age. Nevertheless, in terms of assessing dynamic changes in astigmatism, separate estimations of the cylinder power and meridian must be insufficient.

To solve the problem, we used the Alpins vector analysis as recommended in the Journal of Refractive Surgery. [21–23] Table 3 summarizes the mean polar values of the subjects, which indicate the presence of refractive and keratometric orthogonal astigmatism. Both refractive and keratometric values decreased with age during all three phases. Namely, astigmatism in the whole eye and cornea shifted toward ATR in our cross-sectional investigation, consistent with previous studies [10–17]. Longitudinal investigations also revealed time-dependent decreases in both values in the groups of subjects aged from 40s to 60s. Although these findings may have been caused by the small size of the subpopulation, the astigmatic shift toward ATR appeared to start significantly after the subjects reached 40 years of age. Previous findings also supported a general shift from a predominance of WTR astigmatism to a predominance of ATR astigmatism in adults older than 40 years [12,13,15,16]. The age-related shift towards ATR was not seen in subjects aged ≥ 70 years. Although the cause of these results is unclear, it is possible that they might have been influenced by cataract progression in the refractive value. Data from previous studies revealed that cataracts cause changes in orthogonal astigmatism, which depend on the severity and type of the cataract [31,32] Díez Ajenjo *et al*. [32] reported that nuclear cataracts or posterior subcapsular cataracts may lead to increased WTR astigmatism. Although our data showed that both the refractive and keratometric astigmatism shifted toward ATR from phase 1 to phase 3, the mean polar changes did not differ among two consecutive periods, i.e. from phase 1 to phase 2 or from phase 2 to phase 3 (Table 4). These findings indicate that the shift toward ATR may progress continually and that they did not accelerate with age, at least in periods examined in this research.

Previous data have shown that the shift towards ATR is due to a change in corneal curvature [11,12–14,33], but the details of the underlying mechanism remain unclear. Some reports have presented evidence suggesting that age-related changes in corneal physiology may influence the elasticity and rigidity of the cornea. For example, Daxer *et al*. [34] reported that the mean radius, axial period, and intermolecular Bragg spacing of collagen fibrils is greater in subjects older than 65 years than in younger subjects. These changes in collagen fibrils may result from glycation-induced cross-linking of collagen molecules, which was described by Malik e*t al*. [35]. Thickening of Descemet’s membrane may also influence the corneal curvature [33]. Additionally, data from many studies have suggested that eyelid tension may influence the corneal curvature [36–39]. Read *et al*. [36] reported associations between the angle of the eyelid fissure and the corneal cylinder axis and sphere. In addition, Vihlen *et al*. [37] reported that eyelid tension decreases with age, consistent with an astigmatic shift occurring after 40 years of age. Thus, ophthalmologists should be prepared for performing refractive surgeries, considering these changes.

Questions arise, such as whether WTR or ATR is better for vision quality, or if the astigmatic shift from WTR to ATR is favorable. Although previous studies were conducted in attempt to answer these questions, they showed discordant results. Nevertheless, many studies have shown that near vision tends to be better in individuals with low-myopic ATR astigmatism than in those with WTR astigmatism [40–42]. Rhim *et al*. [43] performed an experimental study of pseudophakic eyes. They found that the focus moves nearer in subjects with ATR astigmatism and farther away in subjects with WTR astigmatism, based on eyelid squinting. ATR astigmatism, induced by aging, may play a role in near vision in the elderly.

Our study provides a basis for considering age-related changes in visual function and astigmatic changes in the years following refractive surgery. This study had strengths compared to previous reports. First, previous reports included mostly cross-sectional studies, whereas our study included a longitudinal investigation, enabling us to investigate age-related changes observed in the same individual over time. Second, we evaluate astigmatism using vector analyses. This allowed us to assess dynamic changes in astigmatism over a period. Our study also had some limitations. First, due to some limitations in the patient histories, we were unable to exclude some conditions. Except for the conditions revealed by slit-lamp or other ophthalmological examinations, we had no choice but to depend on taking the medical histories of patients (e.g. habitual use of contact lenses, eye drops). Second, some unmeasured factors may be potentially associated with astigmatism, such as sleeping habits, the frequency of near work, or biological parameters not included in serological tests. Third, the data obtained by corneal topography or tomography were not included in present study. To gain more detailed information of astigmatism. Although we, and other researchers have reported cross-sectional data [29,44], longitudinal studies are expected.

In conclusion, astigmatism of the cornea and whole eye shifted toward ATR with increasing age. Therefore, we should pay attention to the shift that occurs after refractive surgeries, including cataract surgeries using multifocal or toric intraocular lenses. Our results provide baseline data for the maintenance and improvement of visual function, especially in elderly individuals. Further studies are needed to understand dynamic astigmatic changes better, and a wider range of data collected over an extended period of time will help in improving the maintenance of vision quality.

## References

[1] Morlet N, Minassian D, Dart J. Astigmatism and the analysis of its surgical correction. Br J Ophthalmol. 2001;85: 1127–1138. doi: 10.1136/bjo.85.9.1127 11520769 PMC1724117

[2] Sawides L, Marcos S, Ravikumar S, Thibos L, Bradley A, Webster M. Adaptation to astigmatic blur. J Vis. 2010;10: 22. 10.1167/10.12.22 PMC3244829 21047754

[3] Remón L, Monsoriu JA, Furlan WD. Influence of different types of astigmatism on visual acuity. J Optom. 2017;10: 141–148. 27639497 10.1016/j.optom.2016.07.003 PMC5484781

[4] Artal P, Guirao A. Contributions of the cornea and the lens to the aberrations of the human eye. Opt Lett. 1998;23: 1713–1715. doi: 10.1364/ol.23.001713 18091893

[5] Dubbelman M, Sicam VA, Van der Heijde GL. The shape of the anterior and posterior surface of the aging human cornea. Vision Res. 2006;46: 993–1001. 16266736 10.1016/j.visres.2005.09.021

[6] Katz J, Tielsch JM, Sommer A. Prevalence and risk factors for refractive errors in an adult inner city population. Invest Ophthalmol Vis Sci. 1997;38: 334–340. 9040465

[7] Cheng CY, Hsu WM, Liu JH. Tsai SY, Chou P. Refractive errors in an elderly Chinese population in Taiwan: the Shihpai Eye Study. Invest Ophthalmol Vis Sci. 2003;44: 4630–4638. doi: 10.1167/iovs.03-0169 14578378

[8] Raju P, Ramesh SV, Arvind H, George R, Baskaran M, Paul PG, et al. Prevalence of refractive errors in a rural South Indian population. Invest. Ophthalmol Vis Sci. 2004;45: 4268–4272. doi: 10.1167/iovs.04-0221 15557431

[9] Guzowski M, Wang JJ, Rochtchina E, Rose KA, Mitchell P. Five-year refractive changes in an older population: the Blue Mountains Eye Study. Ophthalmology 2003;110: 1364–1370. doi: 10.1016/S0161-6420(03)00465-2 12867393

[10] Sawada A, Tomidokoro A, Araie M, Iwase A, Yamamoto T; Tajimi Study Group. Refractive errors in an elderly Japanese population: the Tajimi Study. Ophthalmology 2008;115: 363–370. doi: 10.1016/j.ophtha.2007.03.075 18243904

[11] Liu Y C, Chou P, Wojciechowski R, Lin PY, Liu CJ, Chen SJ, et al. Power vector analysis of refractive, corneal, and internal astigmatism in an elderly Chinese population: the Shihpai Eye Study. Invest Ophthalmol Vis. Sci. 2011;52: 9651–9657. doi: 10.1167/iovs.11-7641 22110083

[12] Sanfilippo PG, Yazar S, Kearns L, Sherwin JC, Hewitt AW, Mackey DA. Distribution of astigmatism as a function of age in an Australian population. Acta Ophthalmol. 2015;93: e377–e385. 25585855 10.1111/aos.12644

[13] Baldwin WR, Mills DA. longitudinal study of corneal astigmatism and total astigmatism. Am J Optom Physiol Opt. 1981;58: 206–210. doi: 10.1097/00006324-198103000-00004 7223852

[14] Hayashi K, Hayashi H, Hayashi F. Topographic analysis of the changes in corneal shape due to aging. Cornea 1995;14: 527–532. 8536468

[15] Namba H, Kawasaki R, Sugano A, Murakami T, Nishitsuka K, Kato T, et al. Age-related changes in ocular aberrations and the Yamagata Study (Funagata). Cornea 2017;36: S34–S40. doi: 10.1097/ICO.0000000000001386 28937417

[16] Rozema JJ. Hershko S, Tassignon MJ; for EVICR.net, Project Gullstrand Study Group. The components of adult astigmatism and their age-related changes. Ophthalmic Physiol Opt. 2019;39: 183–193. doi: 10.1111/opo.12616 30994201

[17] Namba H, Kawasaki R, Sugano A, Nishi K, Murakami T, Nishitsuka K, et al. Cross-sectional and longitudinal investigation of the power vector in astigmatism: the Yamagata Study (Funagata). Cornea 2018;37: 53–58. doi: 10.1097/ICO.0000000000001418 29095754

[18] Jaffe NS, Clayman HM. The pathophysiology of corneal astigmatism after cataract extraction. Trans Am Acad Ophthalmol Otolaryngol. 1975;79: OP615–OP630.

[19] Naeser K. Conversion of keratometer readings to polar values. J. Cataract Refract. Surg. 1990;16: 741–745. doi: 10.1016/s0886-3350(13)81018-8 2258811

[20] Thibos L. N., Wheeler W. & Horner D. Power vectors: an application of Fourier analysis to the description and statistical analysis of refractive error. Optom Vis Sci. 1997;74: 367–375. doi: 10.1097/00006324-199706000-00019 9255814

[21] Alpins NA. Astigmatism analysis by the Alpins method. J Cataract Refract Surg. 2001;27: 31–49. doi: 10.1016/s0886-3350(00)00798-7 11165856

[22] Alpins NA. Vector analysis of astigmatism changes by flattening, steepening, and torque. J Cataract Refract Surg. 1997;23: 1503–1514. doi: 10.1016/s0886-3350(97)80021-1 9456408

[23] Reinstein DZ, Archer TJ, Randleman JB. JRS standard for reporting astigmatism outcomes of refractive surgery. J Refract Surg. 2014;30: 654–659. doi: 10.3928/1081597X-20140903-01 25291747

[24] Tominaga M, Eguchi H, Manaka H, Igarashi K, Kato T, Sekikawa A. Impaired glucose tolerance is a risk factor for cardiovascular disease, but not impaired fasting glucose. The Funagata Diabetes Study. Diabetes Care 1999;22: 920–924. doi: 10.2337/diacare.22.6.920 10372242

[25] Kawasaki R, Wang JJ, Rochtchina E, Taylor B, Wong TY, Tominaga M, et al. Cardiovascular risk factors and retinal microvascular signs in an adult Japanese population: the Funagata Study. Ophthalmology 2006;113: 1378–1384. doi: 10.1016/j.ophtha.2006.02.052 16877076

[26] Tanabe Y, Kawasaki R, Wang JJ, Wong TY, Mitchell P, Daimon M, et al. Angiotensin-converting enzyme gene and retinal arteriolar narrowing: the Funagata Study. J Hum Hypertens. 2009;23: 788–793. doi: 10.1038/jhh.2009.27 19369957 PMC2834325

[27] Nishitsuka K, Kawasaki R, Kanno M, Tanabe Y, Saito K, Honma K, et al. Determinants and risk factors for central corneal thickness in Japanese persons: the Funagata Study. Ophthalmic Epidemiol. 2011;18: 244–249. doi: 10.3109/09286586.2011.594206 21961514

[28] Namba H, Kawasaki R, Narumi M, Sugano A, Homma K, Nishi K, et al. Ocular higher-order wavefront aberrations in the Japanese adult population: the Yamagata Study (Funagata). Invest Ophthalmol Vis Sci.2015; 56: 90–97. 10.1167/iovs.14-15261 25503451

[29] Namba H, Sugano A, Nishi K, Murakami T, Nishitsuka K, Konta T, et al. Age-related variations in corneal geometry and their association with astigmatism: The Yamagata Study (Funagata). Medicine 2018;97: e12894. 30412088 10.1097/MD.0000000000012894 PMC6221551

[30] Daimon M, Sato H, Sasaki S, Toriyama S, Emi M, Muramatsu M, et al. Salt consumption-dependent association of the GNB3 gene polymorphism with type 2 DM. Biochem Biophys Res Commun. 2008;374: 576–580.21. doi: 10.1016/j.bbrc.2008.07.065 18656447

[31] Pesudovs K, Elliott DB. Refractive error changes in cortical, nuclear, and posterior subcapsular cataracts. Br J Ophthalmol. 2003;87: 964–967. doi: 10.1136/bjo.87.8.964 12881335 PMC1771794

[32] Díez Ajenjo MA, García Domene MC, Peris Martínez C. Refractive changes in nuclear, cortical and posterior subcapsular cataracts. Effect of the type and grade. J Optom. 2015;8: 86–92. doi: 10.1016/j.optom.2014.07.006 25192610 PMC4401830

[33] Faragher RG, Mulholland B, Tuft SJ, Sandeman S, Khaw PT. Aging and the cornea. Br J Ophthalmol. 1997;81: 814–817. doi: 10.1136/bjo.81.10.814 9486017 PMC1722015

[34] Daxer A, Misof K, Grabner B, Ettl A, Fratzl P. Collagen fibrils in the human corneal stroma: structure and aging. Invest Ophthalmol Vis Sci. 1998;39: 644–648. 9501878

[35] Malik NS, Moss SJ, Ahmed N, Furth AJ, Wall RS, Meek KM. Ageing of the human corneal stroma: structural and biochemical changes. Biochim. Biophys. Acta 1992;1138: 222–228. doi: 10.1016/0925-4439(92)90041-k 1547284

[36] Read SA, Collins MJ, Carney LG. The influence of eyelid morphology on normal corneal shape. Invest Ophthalmol Vis Sci. 2007;48: 112–119. doi: 10.1167/iovs.06-0675 17197524

[37] Vihlen FS, Wilson G. The relation between eyelid tension, corneal toricity, and age. Invest Ophthalmol. Vis. Sci. 1983;24; 1367–1373. 6618796

[38] Cadera W, Orton RB, Hakim O. Changes in astigmatism after surgery for congenital ptosis. J Ped Ophthalmol Strabismus 1992;29: 85–88. 1588481 10.3928/0191-3913-19920301-06

[39] Moon NJ, Lee HI, Kim JC. The changes in corneal astigmatism after botulinum toxin-a injection in patients with blepharospasm. J Korean Med Sci. 2006;21: 131–135 (2006). doi: 10.3346/jkms.2006.21.1.131 16479079 PMC2733961

[40] Verzella F, Calossi A. Multifocal effect of against-the-rule myopic astigmatism in pseudophakic eyes. Refract Corneal Surg. 1993;9: 58–61. 8481374

[41] Nagpal KM, Desai C, Trivedi RH, Vasavada AR. Is pseudophakic astigmatism a desirable goal? Indian J Ophthalmol. 2000;48: 213–216. 11217253

[42] Nanavaty MA, Vasavada AR, Patel AS, Raj SM, Desai TH. Analysis of patients with good uncorrected distance and near vision after monofocal intraocular lens implantation. J Cataract Refract Surg. 2006;32: 1091–1097. doi: 10.1016/j.jcrs.2006.03.021 16857493

[43] Rhim JW, Eom Y, Park SY, Kang SY, Song JS, Kim HM. Eyelid squinting improves near vision in against-the-rule and distance vision in with-the-rule astigmatism in pseudophakic eyes: an eye model experimental study. BMC Ophthalmol. 2020;20: 4. doi: 10.1186/s12886-019-1297-5 31898509 PMC6941361

[44] Vitályos G, Kolozsvári BL, Németh G, Losonczy G, Hassan Z, Pásztor D, et al. Effects of aging on corneal parameters measured with Pentacam in healthy subjects. Sci Rep. 2019;9: 3419. doi: 10.1038/s41598-019-39234-x 30833606 PMC6399218

